# Hermaphroditism in the white spot grouper *Epinephelus coeruleopunctatus *(Pisces: Serranidae) harvested from Padang City waters, Indonesia

**DOI:** 10.12688/f1000research.11090.1

**Published:** 2017-03-24

**Authors:** Usman Bulanin, Masrizal Masrizal, Zainal A. Muchlisin

**Affiliations:** 1 Faculty of Fisheries and Marine Sciences, Bung Hatta University, Padang, 25133, Indonesia; 2 Faculty of Animal Sciences, Andalas University, Padang, 25133, Indonesia; 3 Faculty of Marine and Fisheries, Syiah Kuala University, Banda Aceh, 23111, Indonesia

**Keywords:** Epinephelus coeruleopunctatus, Reproduction, Gonad, Grouper Fish Growth

## Abstract

The objective of the present study was to determine the length (mm) for sex transformation of hermaphroditism in white spot grouper *Epinephelus coeruleopunctatus* as a basis for developing breeding technology. Fish sampling was carried out between April and October 2013 in Padang City waters, Indonesia. A total of 56 white spot groupers were recorded during the study; of these 22 were male, 28 female and 6 samples were not recognized regarding sex preference. Sex differentiation was detected at a length of 183 mm, and at this size the fish are female. Sex transformation to male begun to occur at 302 mm total length.

## Introduction

Groupers (family Serranidae) belong to 109 species and 11 genus ^1, 2^. Groupers are commercial marine fishes that have been harvested intensively from the wild, resulting in decreasing the population worldwide ^3, 4^. The white spot grouper, *Epinephelus coeruleopunctatus,* is one of the most popular groupers and has a high economic value among groupers in Asia-Pacific regions ^5, 6^. However, this species is rare and difficult to catch. According to local fishermen of Padang City, Indonesia, the population of *E. coeruleopunctatus* has been declining sharply over the last two decades ^7^. According to Teixeira *et al*. ^8^ and Mariskha and Abdulgani ^9^ the decreasing fish population is caused by overfishing, habitat perturbation ^10^ and unfriendly fishing practices ^11^. The International Union for Conservation of Nature ^12^ reports this species on the Red List as a threatened species.

Culturing of white spot grouper has been initiated in Indonesia; however, the fry (juveniles) are strongly dependent from the wild supply ^13^. Therefore, it is very crucial to develop breeding technology of the white spot grouper. One of the problems in the development of breeding technology is hermaphroditism sex development, which is observed in this species ^14^. Therefore, it is difficult to determine the sex differentiation between male and female. Hermaphroditism has also been reported in several other groupers, such as *E. tauvina* ^15^, *E. aeneus* ^16^, *E. rivulatus* ^17^, *E. striatus* ^18^, and *Plectropomus laevis* ^19^. Hence, this paper reports on the size (length and body weight) of sex transformation in white spot grouper. This information is crucial to plan a better management strategy of fishery resources ^20^ and to develop breeding technology for the white spot grouper.

## Methods

**Figure 1.**
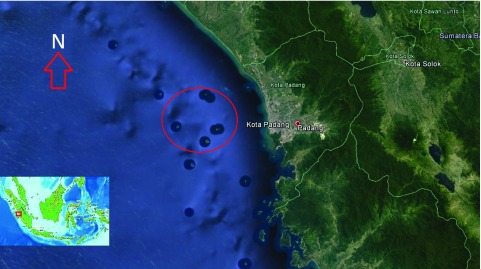
A map of Padang City waters showing the sampling location (red circle) ^7^.

All procedures involving animals were conducted in compliance with Bung Hatta University Research and Ethics Guidelines, Section on Animal Care and Use in Research. Fish were caught from Padang City waters, at GPS coordinates 0 ^0^ 54’ 55.34” S, 100 ^0^ 10’ 15.49” E ( Figure 1), between April and October 2013. The fish were caught using hooks and hand line at the depth of 30–50 m. Fishing operations were carried out from 6.00 am to 16.00 pm. The sampled fish were anesthetized with MS222, prepared by dissolving 4g of MS222 in 5L tap water ^21^ and then transported to the Laboratory of Fisheries Resources of Bung Hatta University for further analysis. In the laboratory, the fish samples were measured for total length (mm) and body weight (g). The abdomen was dissected and the gonad was removed carefully and cleaned using a tissue paper and then weighed nearest to mg using a digital balance (ACIS: AD300; errors 0.01g). Sex differentiation by gonad was examined microscopically (100x magnification) and determined based on Muchlisin *et al*. ^22^. The data were analyzed descriptively.

## Results

A total of 56 fish were recorded during the study, where 50 fish were recognized regarding sex differentiation by gonad, of which 22 were males and 28 females. A total of 6 samples were not recognized regarding their sex, due to being still in the early gonadal development stage. The sex ratio was 2:3 (male:female). The total length of the male fish ranged from 302–537 mm, while females ranged from 183–537 mm. The body weight ranged between 374–2107 g and 85–373 g for male and female fish, respectively. The total length of fish with undetermined sex ranged from 125–242 mm and 85–373 g body weight ( Table 1 and Table 2).

The study showed that the first sex differentiation of *E. coeruleopunctatus* occurred at a size above 183 mm; fish of this size were recognized as female and no male fish were detected in this size group. First sex differentiation is species dependent; for example, *E. bleekeri* occurrs at 170 mm ^23^ and *Plectropomus laevis* at 280 mm ^19^.

**Table 1.** Length frequency distribution of fish *Epinephelus coeruleopunctatus.*

Length class (mm)	N (%)		
	Male	Female	Unrecognized sex
125–182	-	-	3 (50.0)
183–242	-	4 (14.3)	3 (50.0)
243–301	-	4 (14.3)	-
302–360	2 (9.1)	5 (17.9)	-
361–419	6 (27.3)	7 (25.0)	-
420–478	5 (22.7)	5 (17.9)	-
479–537	9 (40.9)	3 (10.7)	-
Total	22 (100)	28 (100)	6 (100)

**Table 2.** Weight frequency distribution of fish *Epinephelus coeruleopunctatus*.

Weight class (g)	N (%)		
	Male	Female	Unrecognized sex
85–372	-	7 (25.0)	6 (100)
374–662	1 (4.5)	7 (25.0)	-
663–951	6 (27.3)	5 (17.9)	-
952–1240	2 (9.1)	2 (7.1)	-
1241–1529	4 (18.2)	2 (7.1)	-
1530–1818	3 (13.6)	5 (17.9)	-
1819–2107	6 (27.3)	-	-
Total	22 (100)	28 (100)	6 (100)

The results revealed that the female white spot grouper begun to transform to male at 302 mm in length, indicating a protogynous hermaphroditism. However, the size at which all fish transform to male fish was unknown, since there were no fish sample more than 537 mm in length. But, the existing data show that the ratio of male fish was increased as total length increased; hence, we suspect that all fish have changed sex to male at sizes above 600 mm. For comparison, Renones *et al*. ^24^ reported that the female dusky grouper *E. marginatus* transforms its sex initially from female to a male at a size of 680 mm and all males were detected at size 800 mm. In addition, Tan and Tan ^25^ reported that *E. tauvina* begins to transform their sex from female to male at the size of 650 mm, while at the size of 700 mm all fish are recognized as male. According to Burhanuddin and Fami ^26^ the occurrence of sex transformation in hermaphroditic fish is species dependent and strongly influenced by environmental factors.

The total length, body weight and sexes of the 56 individual fish sampled Click here for additional data file. Copyright: © 2017 Bulanin U et al. 2017 Data associated with the article are available under the terms of the Creative Commons Zero "No rights reserved" data waiver (CC0 1.0 Public domain dedication).

## Conclusions

The white spot grouper *Epinephelus coeruleopunctatus* is a protogynous hermaphroditism. Sex differentiation was detected at the total length of 183 mm and at this size the fish are female. The sex transformation began to occur at 302 mm total length.

## Data availability

The data referenced by this article are under copyright with the following copyright statement: Copyright: © 2017 Bulanin U et al.

Data associated with the article are available under the terms of the Creative Commons Zero "No rights reserved" data waiver (CC0 1.0 Public domain dedication). 

Dataset 1: The total length, body weight and sexes of the 56 individual fish sampled. doi, 10.5256/f1000research.11090.d155119 ^27^
