# Supplementary material for: Hermaphroditism in the white spot grouper Epinephelus coeruleopunctatus (Pisces: Serranidae) harvested from Padang City waters, Indonesia
Source: F1000Res. 2017 Mar 24;6:320. [Version 1] doi: 10.12688/f1000research.11090.1 (PMC5399959; doi:10.12688/f1000research.11090.1)
Supplement: Peer review history

# Peer review: Referee response for version 1

**DOI:** 10.5256/f1000research.11962.r21903

**Published:** 2017-04-18

**Authors:** Rudy Agung Nugroho

**Affiliations:** 1 Department of Biology, Faculty of Mathematics and Natural Sciences, Mulawarman University, Samarinda, Indonesia

Review report for article entitle “Hermaphroditism in the white spot grouper *Epinephelus coeruleopunctatus *(Pisces: Serranidae) harvested from Padang City waters, Indonesia”. Overall, this article has displayed originality in the work and the outcome of this work adds benefit to the area of the research. This article is presented well with cohesiveness. However, editorial suggestions should be addressed by the author. Below, the basis for suggestion followed by some special editorial concern by section.

  

1. Title:
  The title is short, informative and well constructed.
2. Abstract:
  Suggestion:  Information regarding on how sex differentiation was performed should be written.
3. Introduction:
  The introduction has provided quality relevant information particularly with regard to hermaphroditism. Suggestion: The introduction required to include relevant information specific to sex differentiation by using gonad identification followed by relevant references.
4. Methods:
  a) Please provide specific instrument that used to measure the total length and body weight.
  b) Gonad measurement was performed but there is no gonad weight data in the result section.
  c) Microscopic observations of the gonad is not clearly describe.
  d) "The data were analyzed descriptively" the statement is not clear which data referring to in this article. Please be specific
5. Results:
  a) Length and weight frequency distribution class in the table 1 and 2 is not clearly defined.
  b) Set data or the number of the sample is too small.
  c) It is stated that the occurrence of sex transformation in hermaphroditic fish is species dependent and strongly influenced by environmental factors. The author should include the environment report in the study area. This environment report will give another perspective on this study.
6. Conclusion:
  To conclude the finding that the female differentiated at 183mm and sex changed to male occurred at 302 mm is too “early” because is based on the length and weight. It is better to include histological, endocrinological or event molecular study in this article.
7. References:
  Suggestion: delete ref #27

I have read this submission. I believe that I have an appropriate level of expertise to confirm that it is of an acceptable scientific standard.

# Peer review: Referee response for version 1

**DOI:** 10.5256/f1000research.11962.r21782

**Published:** 2017-04-11

**Authors:** Bradley J. Pusey

**Affiliations:** 1 Centre of Excellence in Natural Resource Management, The University of Western Australia, Perth, WA, Australia

Title and abstract both appropriate.

 Article content – design, methods and analysis all appropriate

 Conclusions are sensible and balanced.  The authors might consider expressing maturation in terms of the length at which 50% of the sample are one sex or another, in addition as say minimum length.  Perhaps more needs to be said of the fact that not all fish in the very largest size class had changed sex into males.

I have read this submission. I believe that I have an appropriate level of expertise to confirm that it is of an acceptable scientific standard.

# Peer review: Referee response for version 1

**DOI:** 10.5256/f1000research.11962.r21264

**Published:** 2017-03-29

**Authors:** Ambok Bolong Abol-Munafi

**Affiliations:** 1 Institute of Tropical Aquaculture, Universiti Malaysia Terengganu, 21030 Kuala Nerus, Terengganu, Malaysia

1. Title

     The title is suitable. Only the word 'harvested' should be deleted.

 2. Abstract

    Suggestion:  A total of 56 white spot groupers were sampled; of these 22 were male, 28 were female and 6 were undifferentiated.

 3. Introduction

     Accepted but the English Language needs to be revised.

 4. Methods

     i)   Please provide the instrument used to measure the total length and body weight.

     ii)  Method for gonad measurement can be removed since the data is not tabled in this article.

     iii) Must have explanation on gonad structure/conditions of functional male and functional female based on microscopic observations that were used for sex determination. I cannot find the explanation in Muchlisin *et al*. (2010) ^1^. Please elaborate on "The data were analyzed descriptively" or the statement is not relevant in this article.

     iv) Please explain on what basis the length class and weight class were decided. Why the differences are not equal. Please recalculate.

 5. Results

   i) The first sentence need to be rephrased.

  ii) Regarding my comment on what basis the length class was decided, the article suggested that the female differentiated at 183mm. If you refer to your Dataset 1, the smallest female is 22.3cm (or 223 mm). Based on the length class, the article suggested that the sex change from female to male occurred at 302 mm. Your Dataset 1 showed that the smallest male caught was 35.0cm (or 350 mm). Please elaborate your data.

 6. Conclusions

 - The sample size is too small and the duration of the study is too short to conclude the finding that the female differentiated at 183mm and sex changed to male occurred at 302 mm.

 7. References

 - No 27 is considered as one of the references?

 General remark:

 - The English language must be revised. Standard terminology should be used, eg: sex change instead of sex transform; differentiated instead of recognized.

I have read this submission. I believe that I have an appropriate level of expertise to confirm that it is of an acceptable scientific standard, however I have significant reservations, as outlined above.

## References

[1] MuchlisinZAMusmanMAzizahMN: Spawning seasons of Rasbora tawarensis (Pisces: Cyprinidae) in Lake Laut Tawar, Aceh province, Indonesia. *Reprod Biol Endocrinol*.2010;8: 10.1186/1477-7827-8-49 49 10.1186/1477-7827-8-49 20482777PMC2887878
